# Resection for malignant tumors in the elbow and individualized reconstruction under assistance of 3D printing technology: A case report

**DOI:** 10.1097/MD.0000000000034854

**Published:** 2023-09-01

**Authors:** Guang-Jun Liao, Qing Su, Yong-Quan Zhang, Li-Ming Chang

**Affiliations:** a Department of Orthopedic Oncology, Yantaishan Hospital, Yantai, Shandong, China; b Yantai Key Laboratory for Repair and Reconstruction of Bone & Joint, Yantai, Shandong, China.

**Keywords:** 3D printing technology, case report, elbow joint replacement, Elbow malignancy, individualized reconstruction

## Abstract

**Rationale:**

With a high failure rate and multiple postoperative complications, the resection for tumors in the elbow and reconstruction present a formidable challenge to orthopedic surgeons. The maturation of 3-dimension (3D) printing technology has facilitated the preoperative design, intraoperative navigation, and reconstruction of bone defects in patients with complex malignant tumors of the elbow joint. In order to improve prognosis, we explored a method of tumor resection and elbow reconstruction aided by 3D printing technology in this research.

**Patient concerns:**

The patient underwent nephrectomy for clear cell carcinoma of the left kidney 3 years ago. Six months ago, the patient presented with limited movement and lateral tenderness in the right elbow joint. The tumor puncture biopsy demonstrated renal clear cell carcinoma metastasis.

**Diagnoses:**

Renal clear cell carcinoma with distal humerus bone metastasis.

**Interventions:**

Thin-layer CT scan data of the patient was acquired, and a 3D reconstruction of both upper limb bones and joints was conducted, followed by a simulation of diseased tissue excision. According to the model, individualized osteotomy guidelines and elbow prostheses were designed and manufactured. Then, prior to the completion of the actual operation, a simulation of the preoperative phase was performed.

**Outcomes:**

The operation was completed without incident. At the 1-, 3-, and 6-month postoperative examinations, both the position and mobility of the prosthesis were found to be satisfactory, and no complications were observed. The hospital for special surgery score and mayo elbow performance score scores increased in comparison to the preoperative period.

**Lessons:**

For patients with complex tumors in the elbow joint, 3D printing technology may assist in the precise excision of the tumor and provide an individualized elbow joint prosthesis that is more precise and effective than traditional surgery. It can accomplish a satisfactory treatment effect for patients when combined with early postoperative scientific rehabilitation training, so it is a method worth promoting.

## 1. Introduction

Both primary malignant tumors and metastatic bone tumors are capable of causing bone destruction,^[1,2]^ and surgical resection of the tumor lesion is essential for long-term survival of patients. Reconstruction after elbow resection due to invasive benign tumors and malignant bone tumors is typically a more challenging issue.^[3,4]^ Although autologous bone replantation is an ideal match, it has a limited purview of application and poor postoperative joint mobility.^[5,6]^ Total elbow arthroplasty is a superior option for patients, but the procedure is more difficult, has a higher failure rate, and is associated with non-negligible complications such as loosening, revision, and infection.^[7–9]^

With the advancement of medical-engineering integration, 3-dimension (3D) printing technology offers a more effective and innovative approach for the clinical treatment of bone malignancies.^[10,11]^ It assists with preoperative design, intraoperative precise navigation, and postoperative individualized reconstruction for complex malignant tumor surgery.^[11,12]^ The application of 3D printing technology in elbow reconstruction surgery could decrease the load on the prosthesis, extend its lifespan, and maximize the restoration of joint function.^[13]^ However, experience with elbow reconstruction guided by 3D printing technology remains limited. Therefore, we describe a novel surgical procedure performed on a patient with distal right humerus metastatic renal cell carcinoma.

## 2. Case presentation

### 2.1. Ethical statement

Our study was involved in elbow joint prosthesis implantation based on 3D printing technology. All study procedures and surgical protocols were sanctioned by the Yantaishan Hospital Medical Ethics Society and conform to Helsinki declaration. The patient was also completely apprized about the publication of this case report and its accompanying photographs. Consent in writing was obtained from the patient.

### 2.2. Clinical data

**Figure 1.**
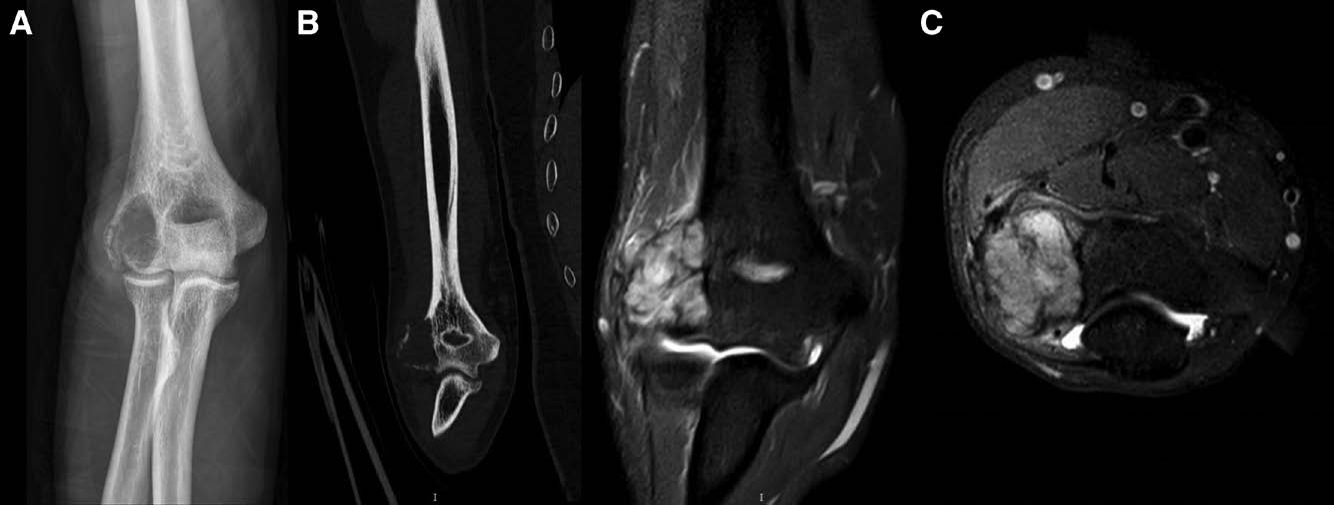
Imaging examination before operation. (A) X-ray, (B) CT, and (C) MRI.

The patient, a 56-year-old man with a height of 176 centimeters and a weight of 68 kilograms, was admitted to the hospital with 6 months of discomfort and limited motion in the lateral right elbow joint. He had left nephrectomy for clear cell renal cell carcinoma of the left kidney 3 years ago. The patient had slight swelling on the lateral side of the right elbow joint, normal skin color and temperature, no visible soft tissue masses, positive local pressure pain on the lateral side of the right elbow joint. The flexion and extension range of motion of the elbow joint was 45° to 80°, pronation 30° and supination 20°. The hospital for special surgery score was 15 and Mayo elbow performance score was 55. Routine X-ray, CT, MRI, whole-body bone scintigram, and PET/CT scans were conducted to confirm that the tumor did not have any additional metastases. A tumor puncture biopsy was conducted on the distal right humerus to elucidate the clear cell renal cell carcinoma bone metastasis (Fig. 1).

### 2.3. Individualized prosthesis and elbow 3D reconstruction

**Figure 2.**
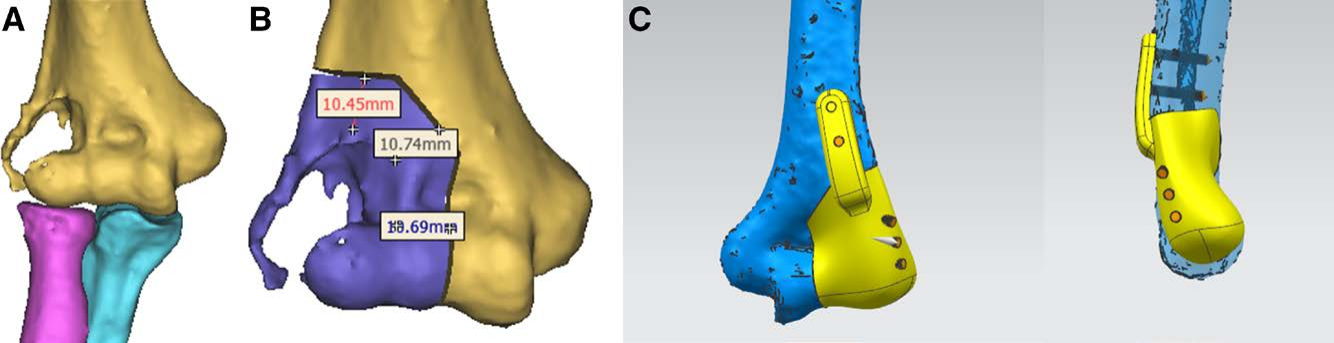
Presurgical 3D reconstruction procedure. (A) 3D depiction of distal humerus tumor, (B) tumor resection of distal humerus, and (C) model of individualized elbow prosthesis. 3D = 3-dimension.

The thin-layer CT scan data was imported into the E3D medical imaging software (http://www.e3d-med.com/, Central South University, Changsha, China) for 3D reconstruction of the bone tissue of the upper limb, which could clearly depict the region of the elbow joint invaded by the tumor (Fig. 2A). On the healthy side of the patient, the same procedure was used to reconstruct the bone tissue model of the elbow joint region. Using the simulated osteotomy function of the E3D software, the tumor-invaded region surrounding the afflicted elbow joint was enlarged by 10mm for a simulation of resection in accordance with the principle of tumor resection (Fig. 2B). Thus, we created models of the elbow joint on the healthy side and the elbow joint on the afflicted side following osteotomy in the form of standard template library file.

In the E3D software, a 3D model of the elbow joint after tumor resection was used to extricate a 10 mm-wide surface along the osteotomy line on the surface of the normal bone tissue at the resection margin and to thicken the body by 3mm. Following the smoothing and optimization of the surface, a 3mm diameter hole was drilled for intraoperative kerf pin fixation. For intraoperative tumor resection, a 3D-printed nylon osteotomy guide plate was autoclaved and used to designate the osteotomy line.

**Figure 3.**
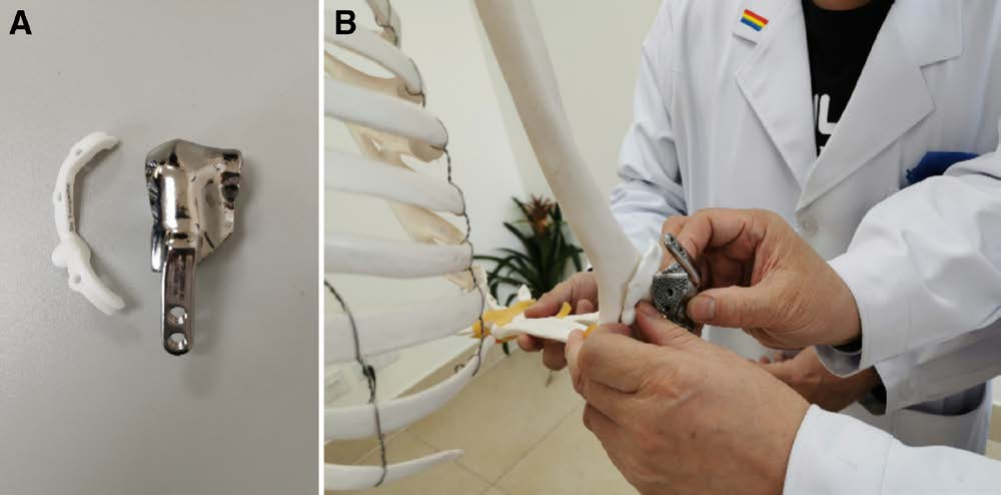
preoperative design and rehearsal. (A) The navigation template and individual elbow prosthesis, (B) exercise in preoperative simulation.

The 3D model of the afflicted elbow joint after osteotomy was imported into UNIGRAPHICS software (UG, Siemens NX 10.0), and the geometry of the patient’s prosthesis for repairing bone defects after elbow osteotomy was designed using the healthy side model as a reference (Fig. 2C). The actual joint reconstruction had to take into account the articular cartilage surface, so the articular surface of the individualized elbow joint prosthesis mentioned above was flared by 1mm. The surface of the elbow joint prosthesis model was polished, the external fixation plate was added, and the interface between the prosthesis and the adjacent bone tissue was designed as a porous structure with a 1.5mm thickness. The elbow prosthesis (Fig. 3A) was fabricated using the selective electron beam melting machine (Arcam A1, Sweden). Then, it was used for presurgical simulation (Fig. 3B).

### 2.4. Operative procedures

**Figure 4.**
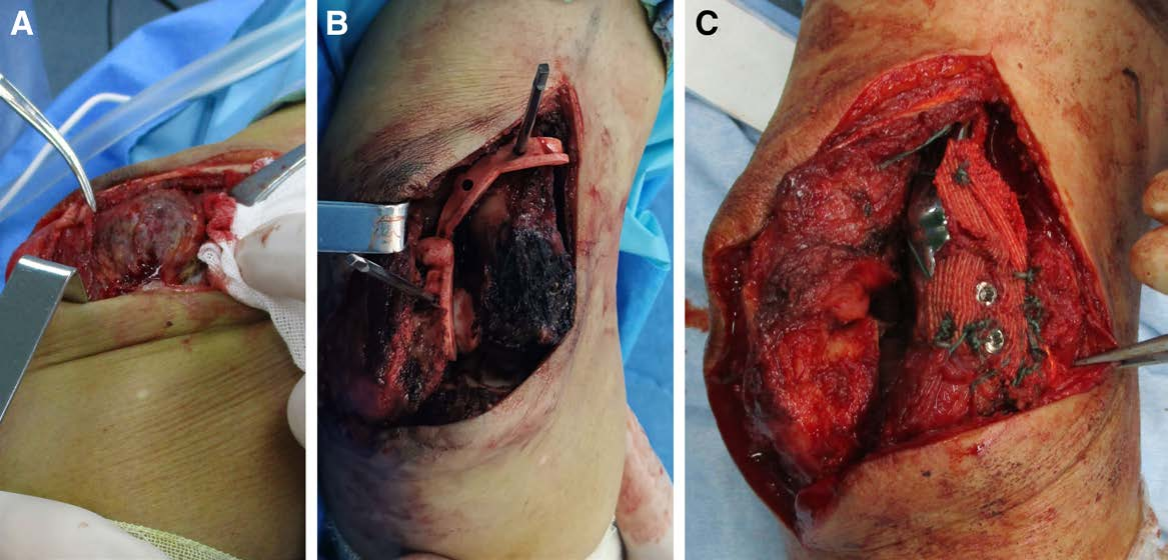
Surgical procedure. (A) exposing the tumor, (B) installing the guide plate and removing the tumor, and (C) installing the prosthesis and artificial patch.

A curved incision was made on the posterior and lateral side of the right elbow joint and extended proximally approximately 5cm above the lateral humeral condyle. The subcutaneous tissue was separated, the fascial layer was cut, the tumor was peeled off, and the common extensor tendon and elbow muscle were severed and marked with a thread for reconstruction of the soft tissue around the elbow joint. After full debridement of the tumor, the triceps muscle was pulled with a pull hook to expose the posterior aspect of the distal humerus and the hawkbill fossa. Additionally, a portion of the joint capsule was removed, and the forearm was externally rotated and the humeroulnar joint was semi-dislocated to expose the lateral humeral condyle. The 3D-printed guide plate was firmly affixed to the posterior region of the distal humerus as well as the fossa of the elbow, secured and marked with a kerf pin. Subsequently, a “C” shaped osteotomy was performed utilizing a swing saw, along the aforementioned mark, to achieve complete resection of the bone segment containing the tumor. After encircling the artificial patch around the surface of the elbow prosthesis and securing it with sutures, the prosthesis was installed in the area of the bone defect and screws were inserted into the corresponding nail channels. In the extended position, the joint capsule, elbow muscle, and common extensor tendon were sutured and fixed to the artificial patch. The elbow joint was reconstructed securely, with excellent flexion and extension (Fig. 4).

### 2.5. Outcomes and follow-up

**Figure 5.**
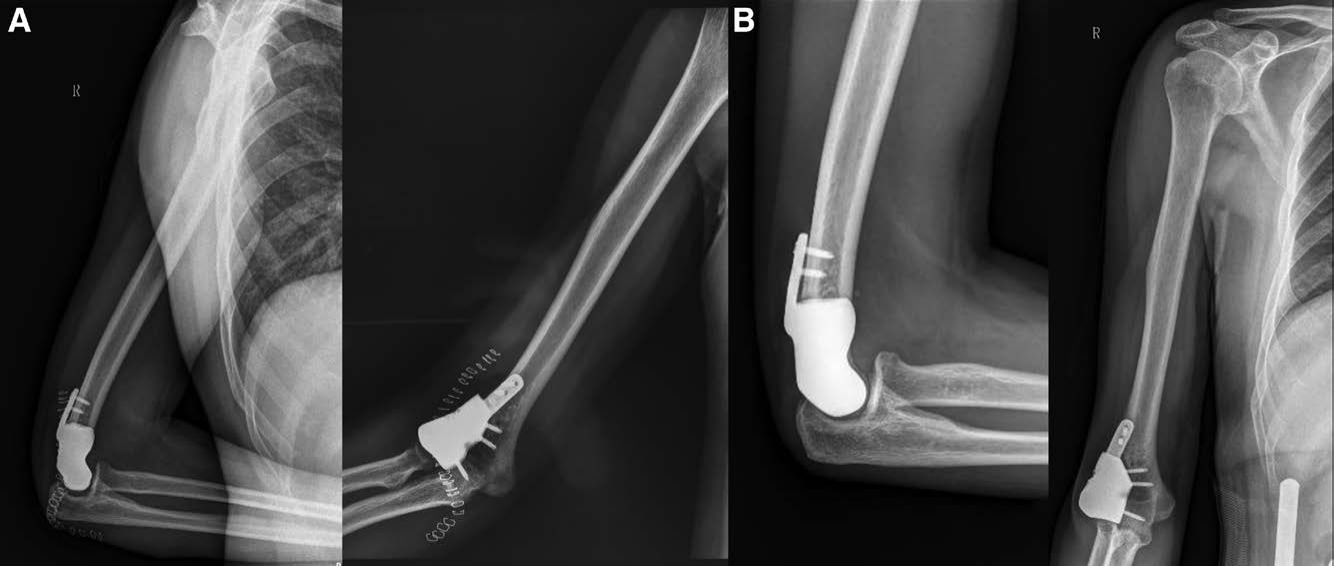
X-rays results of follow-up after operation. (A) Anteroposterior and lateral X-rays one week after surgery, (B) anteroposterior and lateral X-rays six months after surgery.

**Figure 6.**
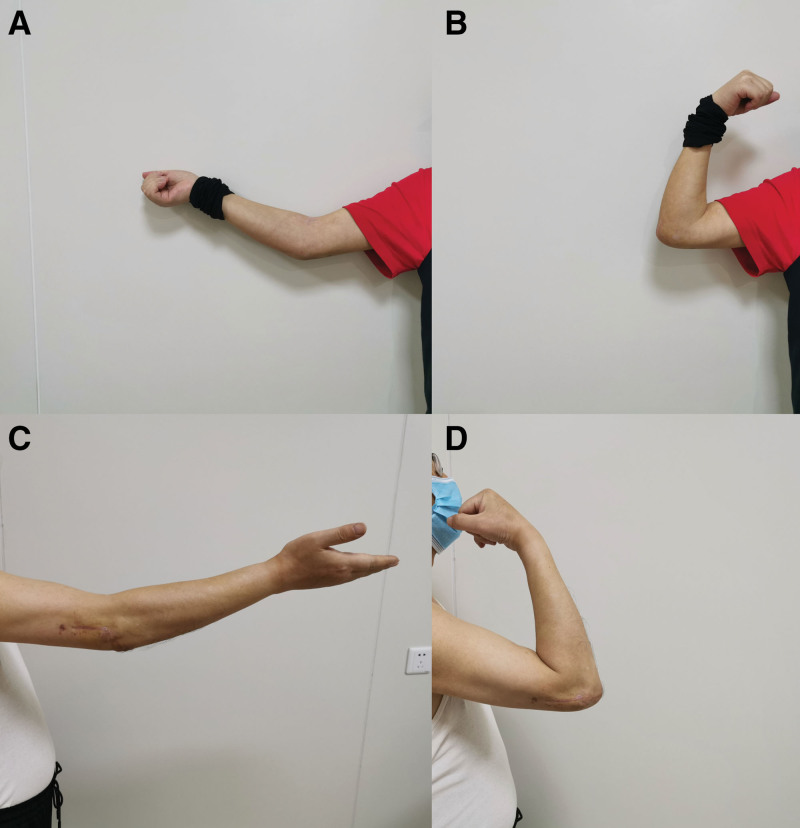
Functional status after operation. (A) extension and, (B) flexion two months after surgery, (C) extension and, and (D) flexion six months after surgery.

With a forearm suspension, the right elbow joint was fixed in a functional position following surgery. On the first postoperative day, the patient was instructed to initiate functional exercise of the affected limb. The drainage tube was removed 2 days after surgery. On the third postoperative day, the swelling around the incision was substantially reduced, the right forearm could be rotated, and the digits and elbow joint could flex and extend independently. One week after surgery, X-rays revealed that the elbow joint prosthesis was in excellent position and conformed to the preoperative design. One month after surgery, finger flexion and extension activities were normal, and the range of elbow flexion and extension activities was 0° to 130°, pronation 70° and supination 70°, which was sufficient for daily activities. In the 1st, 3rd, and 6th months after surgery, the patient returned to the hospital for a follow-up examination and reviewed the elbow joint X-ray (Figs. 5 and 6), which showed the prosthesis in excellent position and angle, with no translucent area or erosion. There were no indications of nerve damage, normal levels of blood sedimentation and C-reactive protein, and no evidence of infection. Table 1 displays the postoperative follow-up mayo elbow performance score and hospital for special surgery score scores.

**Table 1:** HSS and MEPS scores of the patient.

Score	Before operation	After operation (mo)		
		1	3	6
HSS	15	86	90	90
MEPS	55	90	95	95

HSS = hospital for special surgery score, MEPS = mayo elbow performance score.

## 3. Discussion

The principle of surgical treatment for humeral metastases is to provide robust internal fixation as quickly as feasible.^[14]^ Consequently, intramedullary pins, plates, and tumor-type prostheses are frequently employed. However, a significant reduction in joint function is common postoperatively.^[15]^ Due to the complicated anatomy of the elbow joint, total elbow arthroplasty is more difficult and prone to failure, despite the fact that it enhances joint mobility to some degree. The 3D printing technology could precisely reconstruct the anatomical structure of the joint surface with an individualized prosthesis. It not only compensates for the shortcomings of conventional orthopedic surgery, but also facilitates preoperative planning and improves surgeon-patient communication.^[16]^ Spine and pelvic surgeries are increasingly utilizing 3D printing technology, whereas experience with elbow surgery is insufficient.^[17]^

In this case, the patient had extensive bone destruction and limited range of motion in the right elbow joint, but a lengthy survival time. To enhance the postoperative quality of life, we determined to apply 3D printing technology to perform extensive tumor resection and individualized elbow joint reconstruction. Previous researches have demonstrated^[18,19]^ that 3D printing technology simplifies elbow surgery procedures, shortens surgery times, and makes it simpler and more precise to position prostheses compared to conventional surgical methods. The technology of 3D printing places a greater emphasis on the surgeon’s role, enhancing the surgeon’s control over the complete procedure while diminishing the engineer’s contribution. The results of a questionnaire indicated that both physicians and patients were satisfied with 3D printing technology in general.^[20]^

In this example, we recapitulate the benefits of utilizing 3D printing technology as follows: By printing the humeral model, it is able to visualize the tumor. To minimize trauma and reinstate fundamental life functions as soon as possible, the preoperative design preserved the medial humeral condyle, a matching prosthesis was devised, and the anatomical position of elbow joint was restored to the greatest extent feasible; Through preoperative simulation of osteotomy, the preoperative design of a precise osteotomy guide plate assured that the prosthesis matched the osteotomized humerus. By removing tumor tissue in accordance with the guide plate, we accomplished a tumor-free margin for tumor resection and shortened the duration of the operation without intraoperative fluoroscopy; This prosthesis was constructed with an extension plate that was posteriorly attached to the humerus. The alignment of the plate was determined by the humeral anatomy to prevent intraoperative pre-bending of the plate, and the preoperative screw direction and length had been meticulously designed. This substantially reduced the length of time required to restore the skeletal anatomy. However, the follow-up period of this study was brief and it was limited to a single case; its expansion and application must be validated by additional clinical cases and longer-term follow-up results.

## 4. Conclusion

In conclusion, our study effectively treated malignant tumors around the elbow joint using 3D printing technology to assist with precise tumor excision and patient-specific individualization of an elbow prosthesis. In conjunction with early postoperative scientific rehabilitation training, 6-month follow-up of the patient revealed satisfactory treatment outcomes. This research presents a feasible strategy for the individualized treatment of peri-elbow tumors.

## Author contributions

**Conceptualization:** Li-Ming Chang.

**Data curation:** Qing Su, Yong-Quan Zhang.

**Formal analysis:** Qing Su.

**Funding acquisition:** Li-Ming Chang.

**Methodology:** Guang-Jun Liao, Qing Su.

**Resources:** Li-Ming Chang.

**Software:** Guang-Jun Liao, Yong-Quan Zhang.

**Writing – original draft:** Guang-Jun Liao.

**Writing – review & editing:** Guang-Jun Liao, Li-Ming Chang.
